# Crystal structure of 2-{[(naphthalen-1-yl)oxy]meth­yl}-5-(2,4,5-tri­fluoro­phen­yl)-1,3,4-oxa­diazole

**DOI:** 10.1107/S2056989015003205

**Published:** 2015-02-21

**Authors:** Muniyappan Govindhan, Kathavarayan Subramanian, Vijayan Viswanathan, Devadasan Velmurugan

**Affiliations:** a Department of Chemistry, Anna University, Chennai 600 025, India; b Orchid Chemicals & Pharmaceuticals Ltd, R&D Centre, Sholinganallur, Chennai 600 119, India; c Centre of Advanced Study in Crystallography and Biophysics, University of Madras, Guindy Campus, Chennai 600 025, India

**Keywords:** crystal structure, naphthalen-1-yl­oxy, tri­fluoro­phen­yl, 1,3,4-oxa­diazole, hydrogen bonding

## Abstract

In the title compound C_19_H_11_F_3_N_2_O_2_, the oxa­diazole ring and the naphthalene ring system are approximately planar (r.m.s. deviations of 0.001 and 0.020 Å, respectively) and the oxa­diazole ring makes dihedral angles of 13.11 (1) and 7.59 (1)° with the naphthalene ring system and the tri­fluoro­phenyl ring, respectively. In the crystal, C—H⋯N hydrogen bonds link mol­ecules into chains along the *a*-axis direction, while C—H⋯F contacts form additional chains along the *ac* diagonal. These contacts generate sheets of mol­ecules approximately parallel to the (011) plane.

## Related literature   

For the biological activity and other applications of triazole derivatives, see: Desai *et al.* (2014 ▸); Bhat *et al.* (2011 ▸); Katrin *et al.* (2005 ▸); Shailaja *et al.* (2010 ▸).
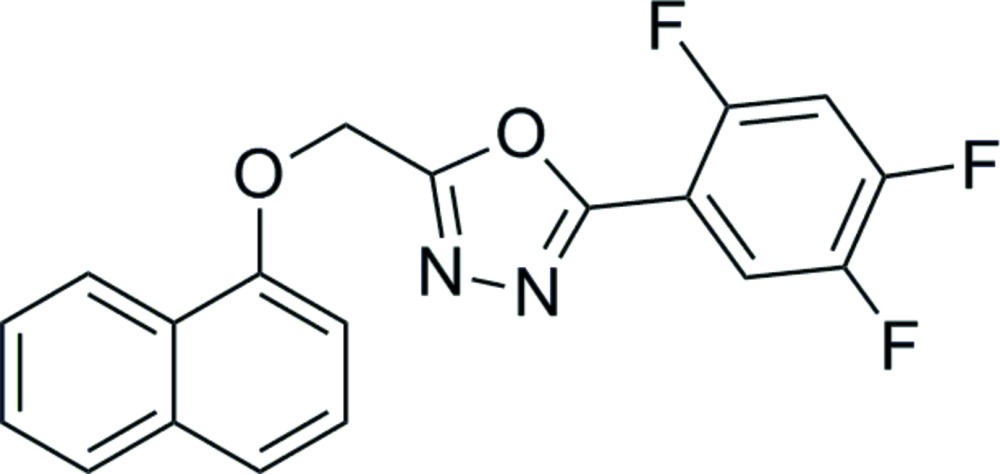


## Experimental   

### Crystal data  

C_19_H_11_F_3_N_2_O_2_
*M* *_r_* = 356.30
Triclinic, 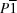

*a* = 7.4817 (5) Å
*b* = 7.5928 (5) Å
*c* = 15.7908 (10) Å
α = 78.673 (3)°
β = 78.404 (3)°
γ = 65.370 (3)°
*V* = 792.36 (9) Å^3^
*Z* = 2
Mo *K*α radiation
μ = 0.12 mm^−1^
*T* = 293 K
0.25 × 0.15 × 0.10 mm

### Data collection  

Bruker SMART APEXII area-detector diffractometer
Absorption correction: multi-scan (*SADABS*; Bruker, 2008 ▸) *T*_min_ = 0.970, *T*_max_ = 0.988
11539 measured reflections
3330 independent reflections
2402 reflections with *I* > 2σ(*I*)
*R*_int_ = 0.031

### Refinement  

*R*[*F* ^2^ > 2σ(*F* ^2^)] = 0.047
*wR*(*F* ^2^) = 0.140
*S* = 1.04
3330 reflections
236 parameters
H-atom parameters constrained
Δρ_max_ = 0.22 e Å^−3^
Δρ_min_ = −0.31 e Å^−3^

### 

Data collection: *APEX2* (Bruker, 2008 ▸); cell refinement: *SAINT* (Bruker, 2008 ▸); data reduction: *SAINT*; program(s) used to solve structure: *SHELXS97* (Sheldrick, 2008 ▸); program(s) used to refine structure: *SHELXL97* (Sheldrick, 2008 ▸); molecular graphics: *ORTEP-3 for Windows* (Farrugia, 2012 ▸); software used to prepare material for publication: *SHELXL97* and *PLATON* (Spek, 2009 ▸).

## Supplementary Material

Crystal structure: contains datablock(s) global, I. DOI: 10.1107/S2056989015003205/sj5445sup1.cif

Structure factors: contains datablock(s) I. DOI: 10.1107/S2056989015003205/sj5445Isup2.hkl

Supporting information file. DOI: 10.1107/S2056989015003205/sj5445Isup3.cml

**Fig. 1.**
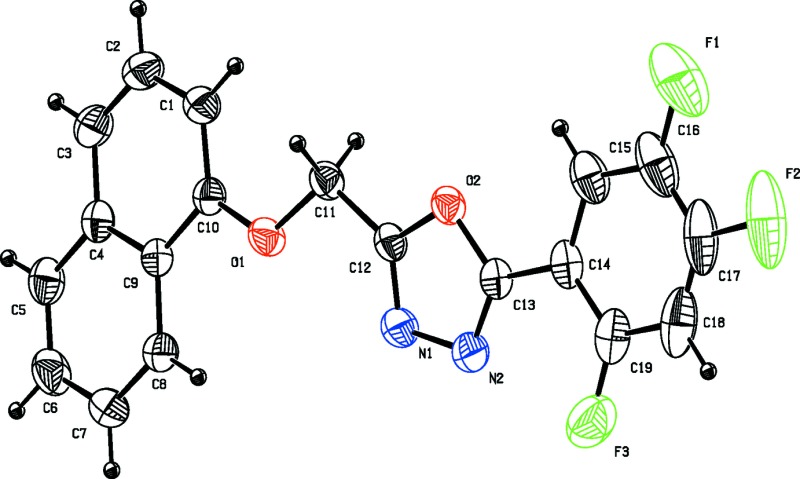
The molecular structure of the title compound, showing the atomic numbering with displacement ellipsoids drawn at the 30% probability level.

**Fig. 2.**
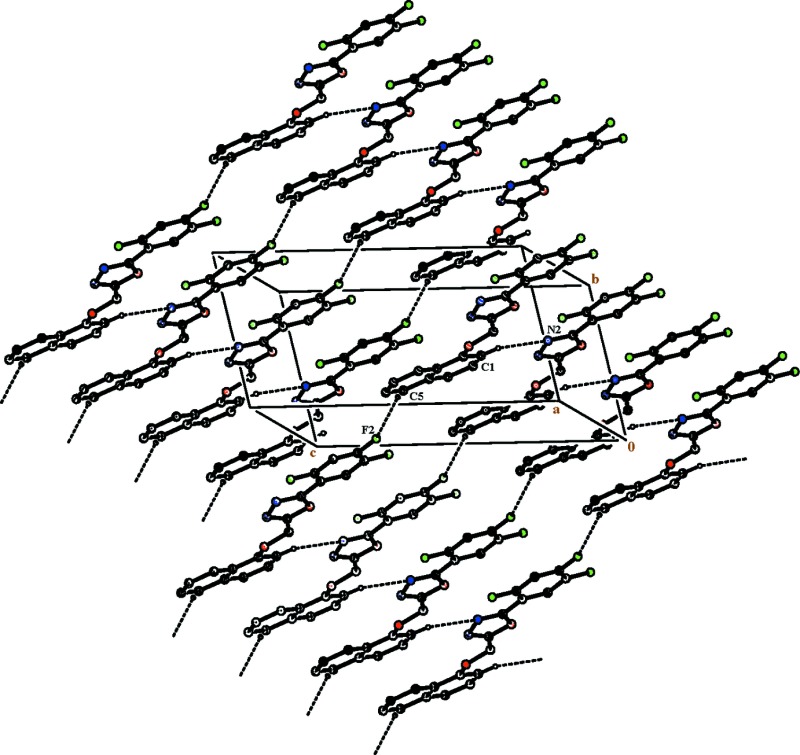
Crystal packing of the title compound viewed along the a axis H-atoms not involved in H-bonds have been excluded for clarity.

. DOI: 10.1107/S2056989015003205/sj5445fig1.tif
The mol­ecular structure of the title compound, showing the atomic numbering with displacement ellipsoids drawn at the 30% probability level.

a . DOI: 10.1107/S2056989015003205/sj5445fig2.tif
Crystal packing of the title compound viewed along the *a* axis H-atoms not involved in H-bonds have been excluded for clarity.

CCDC reference: 1049573

Additional supporting information:  crystallographic information; 3D view; checkCIF report

## Figures and Tables

**Table 1:** Hydrogen-bond geometry (, )

*D*H*A*	*D*H	H*A*	*D* *A*	*D*H*A*
C1H1N2^i^	0.93	2.61	3.449(3)	151
C5H5F2^ii^	0.93	2.51	3.290(3)	141

Symmetry codes: (i) 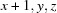; (ii) 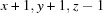.

## supplementary crystallographic information

### S1. Comment

1,3,4-Oxadiazoles are a class of 5-membered heterocyclic compounds that have a wide range of biological activity as well as polymer and material science applications (Shailaja *et al.*, 2010). Their derivatives are commonly used pharmacophores due to their metabolic profile and ability to engage in hydrogen bonding. Their pharmaceutical applications include use as anti-inflammatory, analgesic, anti-HIV, antimycobacterial agents, cathepsin K, tyrosinase and monoamine oxidase (MAO) inhibitors (Desai *et al.*, 2014). They are also used as anticonvulsant, anticancer, antifungal and tuberculostatic drugs (Bhat *et al.*,2011). They also have analgesic, antiplatelet and antithrombotic activities (Katrin *et al.*, 2005). Moreover, their amino derivatives are very commonly used as antimicrobial and germicidal agents.

In the title compound C_19_H_11_N_2_O_2_F_3_, Fig. 1, the oxadiazole, napthalene and trifluorophenyl rings are planar and the oxadiazole ring (C12/N1/N2/C13/O2) makes dihedral angles of 13.11 (1)° and 7.59 (1)° with the naphthalene (C1-C10) ring system and the trifluorophenyl (C14—C19) ring respectively. The fluorine F1, F2 and F3 atoms deviate from the benzene ring by -0.0017 Å, 0.0175 Å and -0.0163 Å respectively.

In the crystal C1–H1···N2 hydrogen bonds link molecules into chains along *a* while C5–H5···F2 contacts form additional chains along the *ac* diagonal. These contacts generate sheets of molecules approximately parallel to the (011) plane. Within these sheets groups of four molecules form R^4^_4_(37) ring motifs.

### S2. Experimental

Iodobenzene diacetate (2.0 mol eq) was added to a solution of naphthalen-1-yloxy-acetic acid (2, 4, 5-trifluoro-benzylidene)-hydrazide (1.0 mole eq) in dioxane (10mL) at 25-30 ° C and stirred at the same temperature for 15-30 minutes. Completion of the reaction was confirmed by TLC (mobile phase ethyl acetate/hexane, 3:7). The dioxane was distilled off under vacuum. The resulting residue was dissolved in ethyl acetate and washed with saturated sodium bicarbonate solution, followed by water and brine solution. The separated organic layer was dried over anhydrous sodium sulfate and distilled under vacuum. The crude product was purified by column chromatography over silica gel (60-120 mesh) using hexane and ethyl acetate (9:1) as eluent to afford the pure product of as an off-white solid. After purification the compound was crystallized from methanol by the slow evaporation method.

### S3. Refinement

The hydrogen atoms were placed in calculated positions with C—H = 0.93Å, refined in the riding model with fixed isotropic displacement parameters: *U*_iso_(H) = 1.5*U*_eq_(C) for the methyl group and *U*_iso_(H) = 1.2*U*_eq_(C) for other H atoms.

### Crystal data

**Table:**

C_19_H_11_F_3_N_2_O_2_	*Z* = 2
*M**_r_* = 356.30	*F*(000) = 364
Triclinic, *P*1	*D*_x_ = 1.493 Mg m^−^^3^
Hall symbol: -P 1	Mo *K*α radiation, λ = 0.71073 Å
*a* = 7.4817 (5) Å	Cell parameters from 3330 reflections
*b* = 7.5928 (5) Å	θ = 1.3–26.8°
*c* = 15.7908 (10) Å	µ = 0.12 mm^−^^1^
α = 78.673 (3)°	*T* = 293 K
β = 78.404 (3)°	Block, colourless
γ = 65.370 (3)°	0.25 × 0.15 × 0.10 mm
*V* = 792.36 (9) Å^3^	

### Data collection

**Table:**

Bruker SMART APEXII area-detector diffractometer	3330 independent reflections
Radiation source: fine-focus sealed tube	2402 reflections with *I* > 2σ(*I*)
Graphite monochromator	*R*_int_ = 0.031
ω and φ scans	θ_max_ = 26.8°, θ_min_ = 1.3°
Absorption correction: multi-scan (*SADABS*; Bruker, 2008)	*h* = −9→9
*T*_min_ = 0.970, *T*_max_ = 0.988	*k* = −9→9
11539 measured reflections	*l* = −19→19

### Refinement

**Table:**

Refinement on *F*^2^	Secondary atom site location: difference Fourier map
Least-squares matrix: full	Hydrogen site location: inferred from neighbouring sites
*R*[*F*^2^ > 2σ(*F*^2^)] = 0.047	H-atom parameters constrained
*wR*(*F*^2^) = 0.140	*w* = 1/[σ^2^(*F*_o_^2^) + (0.062*P*)^2^ + 0.2065*P*] where *P* = (*F*_o_^2^ + 2*F*_c_^2^)/3
*S* = 1.03	(Δ/σ)_max_ < 0.001
3330 reflections	Δρ_max_ = 0.22 e Å^−^^3^
236 parameters	Δρ_min_ = −0.31 e Å^−^^3^
0 restraints	Extinction correction: *SHELXL97* (Sheldrick, 2008), Fc^*^=kFc[1+0.001xFc^2^λ^3^/sin(2θ)]^-1/4^
Primary atom site location: structure-invariant direct methods	Extinction coefficient: 0.006 (2)

### Special details

**Table:**

Geometry. All esds (except the esd in the dihedral angle between two l.s. planes) are estimated using the full covariance matrix. The cell esds are taken into account individually in the estimation of esds in distances, angles and torsion angles; correlations between esds in cell parameters are only used when they are defined by crystal symmetry. An approximate (isotropic) treatment of cell esds is used for estimating esds involving l.s. planes.
Refinement. Refinement of F^2^ against ALL reflections. The weighted R-factor wR and goodness of fit S are based on F^2^, conventional R-factors R are based on F, with F set to zero for negative F^2^. The threshold expression of F^2^ > 2sigma(F^2^) is used only for calculating R-factors(gt) etc. and is not relevant to the choice of reflections for refinement. R-factors based on F^2^ are statistically about twice as large as those based on F, and R- factors based on ALL data will be even larger.

### Fractional atomic coordinates and isotropic or equivalent isotropic displacement parameters (Å^2^)

**Table:**

	*x*	*y*	*z*	*U*_iso_*/*U*_eq_	
C1	0.4888 (3)	0.3315 (3)	0.37814 (13)	0.0588 (5)	
H1	0.5114	0.2802	0.4354	0.071*	
C2	0.6415 (3)	0.3567 (3)	0.31604 (15)	0.0691 (6)	
H2	0.7656	0.3212	0.3326	0.083*	
C3	0.6121 (3)	0.4313 (3)	0.23273 (14)	0.0673 (5)	
H3	0.7168	0.4439	0.1925	0.081*	
C4	0.4239 (3)	0.4907 (3)	0.20584 (12)	0.0543 (5)	
C5	0.3867 (3)	0.5717 (3)	0.12011 (13)	0.0670 (5)	
H5	0.4891	0.5862	0.0789	0.080*	
C6	0.2045 (4)	0.6293 (3)	0.09616 (14)	0.0744 (6)	
H6	0.1830	0.6829	0.0390	0.089*	
C7	0.0489 (3)	0.6080 (3)	0.15737 (14)	0.0699 (6)	
H7	−0.0759	0.6483	0.1406	0.084*	
C8	0.0783 (3)	0.5289 (3)	0.24135 (13)	0.0578 (5)	
H8	−0.0265	0.5158	0.2814	0.069*	
C9	0.2666 (3)	0.4668 (2)	0.26779 (11)	0.0474 (4)	
C10	0.3070 (3)	0.3827 (2)	0.35414 (11)	0.0483 (4)	
C11	0.1842 (3)	0.2579 (3)	0.49334 (11)	0.0540 (4)	
H11A	0.2063	0.3365	0.5285	0.065*	
H11B	0.2998	0.1361	0.4898	0.065*	
C12	0.0033 (3)	0.2197 (2)	0.53195 (11)	0.0491 (4)	
C13	−0.1893 (3)	0.1278 (2)	0.63030 (12)	0.0516 (4)	
C14	−0.2556 (3)	0.0518 (3)	0.71692 (12)	0.0595 (5)	
C15	−0.1309 (4)	−0.0186 (3)	0.78032 (14)	0.0767 (6)	
H15	−0.0047	−0.0168	0.7679	0.092*	
C16	−0.1951 (6)	−0.0916 (4)	0.86218 (16)	0.1020 (10)	
C17	−0.3818 (6)	−0.0941 (4)	0.88135 (19)	0.1061 (12)	
C18	−0.5058 (5)	−0.0245 (4)	0.8207 (2)	0.1034 (11)	
H18	−0.6323	−0.0254	0.8342	0.124*	
C19	−0.4444 (4)	0.0477 (3)	0.73917 (17)	0.0761 (7)	
N1	−0.1442 (3)	0.2435 (2)	0.49547 (10)	0.0622 (4)	
N2	−0.2727 (2)	0.1818 (2)	0.56086 (11)	0.0620 (4)	
O1	0.14915 (18)	0.35881 (19)	0.40929 (8)	0.0582 (4)	
O2	−0.01186 (18)	0.14762 (17)	0.61719 (7)	0.0520 (3)	
F1	−0.0726 (4)	−0.1601 (3)	0.92306 (11)	0.1607 (9)	
F2	−0.4342 (4)	−0.1687 (3)	0.96228 (11)	0.1684 (11)	
F3	−0.5703 (2)	0.1171 (3)	0.68014 (13)	0.1095 (6)	

### Atomic displacement parameters (Å^2^)

**Table:**

	*U*^11^	*U*^22^	*U*^33^	*U*^12^	*U*^13^	*U*^23^
C1	0.0581 (11)	0.0638 (11)	0.0548 (11)	−0.0257 (9)	−0.0118 (9)	0.0004 (9)
C2	0.0548 (11)	0.0827 (14)	0.0737 (14)	−0.0332 (10)	−0.0113 (10)	−0.0018 (11)
C3	0.0564 (11)	0.0796 (13)	0.0672 (13)	−0.0357 (10)	0.0033 (10)	−0.0038 (10)
C4	0.0632 (11)	0.0516 (9)	0.0494 (10)	−0.0280 (8)	0.0000 (8)	−0.0044 (8)
C5	0.0804 (14)	0.0697 (12)	0.0523 (11)	−0.0389 (11)	0.0030 (10)	−0.0017 (9)
C6	0.0996 (18)	0.0763 (14)	0.0483 (11)	−0.0395 (13)	−0.0165 (11)	0.0081 (10)
C7	0.0737 (13)	0.0743 (13)	0.0643 (13)	−0.0317 (11)	−0.0240 (11)	0.0069 (10)
C8	0.0586 (11)	0.0608 (11)	0.0557 (11)	−0.0285 (9)	−0.0075 (9)	0.0000 (8)
C9	0.0538 (10)	0.0431 (8)	0.0461 (9)	−0.0215 (7)	−0.0049 (7)	−0.0038 (7)
C10	0.0498 (10)	0.0478 (9)	0.0468 (10)	−0.0212 (7)	−0.0020 (8)	−0.0047 (7)
C11	0.0594 (11)	0.0543 (10)	0.0452 (10)	−0.0225 (8)	−0.0082 (8)	0.0021 (8)
C12	0.0587 (10)	0.0461 (9)	0.0404 (9)	−0.0205 (8)	−0.0072 (8)	−0.0006 (7)
C13	0.0597 (11)	0.0440 (9)	0.0495 (10)	−0.0213 (8)	−0.0028 (8)	−0.0049 (7)
C14	0.0797 (13)	0.0438 (9)	0.0496 (11)	−0.0250 (9)	0.0047 (9)	−0.0061 (8)
C15	0.1047 (18)	0.0658 (12)	0.0487 (12)	−0.0290 (12)	−0.0033 (11)	−0.0001 (9)
C16	0.170 (3)	0.0668 (14)	0.0494 (13)	−0.0327 (17)	−0.0085 (17)	0.0005 (11)
C17	0.174 (3)	0.0633 (14)	0.0662 (17)	−0.0576 (18)	0.047 (2)	−0.0151 (12)
C18	0.130 (3)	0.0742 (16)	0.100 (2)	−0.0583 (17)	0.0498 (19)	−0.0246 (15)
C19	0.0887 (16)	0.0575 (12)	0.0788 (15)	−0.0364 (11)	0.0182 (13)	−0.0137 (11)
N1	0.0677 (10)	0.0713 (10)	0.0480 (9)	−0.0315 (8)	−0.0122 (8)	0.0060 (7)
N2	0.0668 (10)	0.0677 (10)	0.0549 (10)	−0.0327 (8)	−0.0108 (8)	0.0020 (8)
O1	0.0534 (7)	0.0692 (8)	0.0444 (7)	−0.0241 (6)	−0.0046 (5)	0.0083 (6)
O2	0.0606 (8)	0.0538 (7)	0.0410 (7)	−0.0243 (6)	−0.0080 (5)	0.0010 (5)
F1	0.249 (3)	0.1436 (16)	0.0596 (10)	−0.0549 (17)	−0.0400 (13)	0.0235 (10)
F2	0.303 (3)	0.1016 (12)	0.0759 (11)	−0.1005 (16)	0.0777 (14)	−0.0141 (9)
F3	0.0853 (10)	0.1222 (13)	0.1295 (14)	−0.0566 (9)	0.0005 (10)	−0.0139 (11)

### Geometric parameters (Å, º)

**Table:**

C1—C10	1.361 (3)	C11—C12	1.487 (3)
C1—C2	1.401 (3)	C11—H11A	0.9700
C1—H1	0.9300	C11—H11B	0.9700
C2—C3	1.349 (3)	C12—N1	1.277 (2)
C2—H2	0.9300	C12—O2	1.351 (2)
C3—C4	1.415 (3)	C13—N2	1.283 (2)
C3—H3	0.9300	C13—O2	1.368 (2)
C4—C5	1.404 (3)	C13—C14	1.451 (3)
C4—C9	1.421 (2)	C14—C15	1.381 (3)
C5—C6	1.355 (3)	C14—C19	1.398 (3)
C5—H5	0.9300	C15—C16	1.378 (3)
C6—C7	1.400 (3)	C15—H15	0.9300
C6—H6	0.9300	C16—F1	1.340 (4)
C7—C8	1.364 (3)	C16—C17	1.376 (5)
C7—H7	0.9300	C17—F2	1.344 (3)
C8—C9	1.410 (3)	C17—C18	1.344 (5)
C8—H8	0.9300	C18—C19	1.366 (4)
C9—C10	1.422 (2)	C18—H18	0.9300
C10—O1	1.375 (2)	C19—F3	1.334 (3)
C11—O1	1.411 (2)	N1—N2	1.413 (2)
			
C10—C1—C2	119.51 (18)	C12—C11—H11A	110.5
C10—C1—H1	120.2	O1—C11—H11B	110.5
C2—C1—H1	120.2	C12—C11—H11B	110.5
C3—C2—C1	121.32 (19)	H11A—C11—H11B	108.7
C3—C2—H2	119.3	N1—C12—O2	113.26 (16)
C1—C2—H2	119.3	N1—C12—C11	129.06 (16)
C2—C3—C4	120.79 (18)	O2—C12—C11	117.67 (15)
C2—C3—H3	119.6	N2—C13—O2	112.38 (16)
C4—C3—H3	119.6	N2—C13—C14	129.71 (19)
C5—C4—C3	122.47 (18)	O2—C13—C14	117.91 (17)
C5—C4—C9	118.68 (18)	C15—C14—C19	118.1 (2)
C3—C4—C9	118.85 (18)	C15—C14—C13	120.0 (2)
C6—C5—C4	121.36 (19)	C19—C14—C13	122.0 (2)
C6—C5—H5	119.3	C16—C15—C14	119.4 (3)
C4—C5—H5	119.3	C16—C15—H15	120.3
C5—C6—C7	120.0 (2)	C14—C15—H15	120.3
C5—C6—H6	120.0	F1—C16—C17	120.4 (3)
C7—C6—H6	120.0	F1—C16—C15	118.9 (3)
C8—C7—C6	120.7 (2)	C17—C16—C15	120.7 (3)
C8—C7—H7	119.6	F2—C17—C18	121.7 (4)
C6—C7—H7	119.6	F2—C17—C16	117.6 (4)
C7—C8—C9	120.36 (18)	C18—C17—C16	120.7 (3)
C7—C8—H8	119.8	C17—C18—C19	119.4 (3)
C9—C8—H8	119.8	C17—C18—H18	120.3
C8—C9—C4	118.84 (17)	C19—C18—H18	120.3
C8—C9—C10	123.12 (16)	F3—C19—C18	118.4 (3)
C4—C9—C10	118.04 (16)	F3—C19—C14	119.9 (2)
C1—C10—O1	124.10 (16)	C18—C19—C14	121.7 (3)
C1—C10—C9	121.46 (16)	C12—N1—N2	105.99 (15)
O1—C10—C9	114.45 (15)	C13—N2—N1	106.01 (15)
O1—C11—C12	106.06 (14)	C10—O1—C11	117.48 (14)
O1—C11—H11A	110.5	C12—O2—C13	102.37 (13)
			
C10—C1—C2—C3	0.2 (3)	C13—C14—C15—C16	−179.59 (18)
C1—C2—C3—C4	1.4 (3)	C14—C15—C16—F1	179.8 (2)
C2—C3—C4—C5	179.02 (19)	C14—C15—C16—C17	−0.3 (4)
C2—C3—C4—C9	−1.2 (3)	F1—C16—C17—F2	−0.6 (4)
C3—C4—C5—C6	−179.3 (2)	C15—C16—C17—F2	179.5 (2)
C9—C4—C5—C6	0.9 (3)	F1—C16—C17—C18	179.6 (2)
C4—C5—C6—C7	−0.2 (3)	C15—C16—C17—C18	−0.3 (4)
C5—C6—C7—C8	−0.3 (3)	F2—C17—C18—C19	−179.3 (2)
C6—C7—C8—C9	−0.1 (3)	C16—C17—C18—C19	0.6 (4)
C7—C8—C9—C4	0.9 (3)	C17—C18—C19—F3	−179.6 (2)
C7—C8—C9—C10	−179.79 (17)	C17—C18—C19—C14	−0.2 (4)
C5—C4—C9—C8	−1.3 (3)	C15—C14—C19—F3	179.00 (19)
C3—C4—C9—C8	178.98 (17)	C13—C14—C19—F3	−0.7 (3)
C5—C4—C9—C10	179.36 (16)	C15—C14—C19—C18	−0.4 (3)
C3—C4—C9—C10	−0.4 (2)	C13—C14—C19—C18	179.84 (19)
C2—C1—C10—O1	178.07 (17)	O2—C12—N1—N2	−0.3 (2)
C2—C1—C10—C9	−1.9 (3)	C11—C12—N1—N2	178.92 (17)
C8—C9—C10—C1	−177.39 (17)	O2—C13—N2—N1	−0.1 (2)
C4—C9—C10—C1	2.0 (2)	C14—C13—N2—N1	179.94 (17)
C8—C9—C10—O1	2.7 (2)	C12—N1—N2—C13	0.2 (2)
C4—C9—C10—O1	−177.99 (14)	C1—C10—O1—C11	−6.4 (3)
O1—C11—C12—N1	9.7 (3)	C9—C10—O1—C11	173.59 (14)
O1—C11—C12—O2	−171.20 (14)	C12—C11—O1—C10	−169.31 (14)
N2—C13—C14—C15	172.59 (19)	N1—C12—O2—C13	0.18 (19)
O2—C13—C14—C15	−7.3 (3)	C11—C12—O2—C13	−179.10 (14)
N2—C13—C14—C19	−7.7 (3)	N2—C13—O2—C12	−0.01 (19)
O2—C13—C14—C19	172.39 (16)	C14—C13—O2—C12	179.92 (14)
C19—C14—C15—C16	0.7 (3)		

### Hydrogen-bond geometry (Å, º)

**Table:**

*D*—H···*A*	*D*—H	H···*A*	*D*···*A*	*D*—H···*A*
C1—H1···N2^i^	0.93	2.61	3.449 (3)	151
C5—H5···F2^ii^	0.93	2.51	3.290 (3)	141

Symmetry codes: (i) *x*+1, *y*, *z*; (ii) *x*+1, *y*+1, *z*−1.
