# Supplementary figures and images for: Crystal structure of 2-{[(naphthalen-1-yl)oxy]meth­yl}-5-(2,4,5-tri­fluoro­phen­yl)-1,3,4-oxa­diazole
Source: Acta Crystallogr E Crystallogr Commun. 2015 Feb 21;71(Pt 3):o190–1. doi: 10.1107/S2056989015003205 (PMC4350736; doi:10.1107/S2056989015003205)

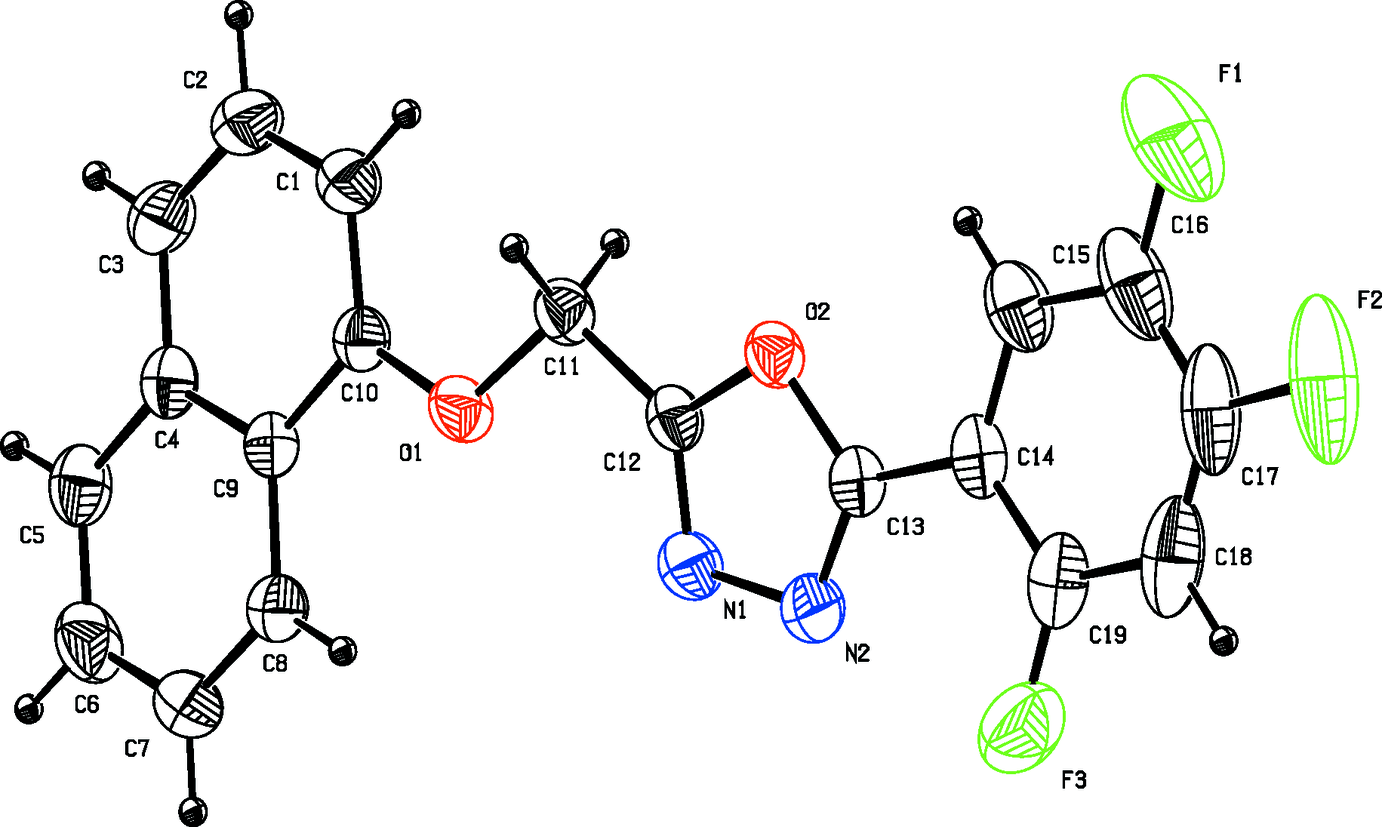

Supplement: Supplementary file 4 [file e-71-0o190-fig1.tif]

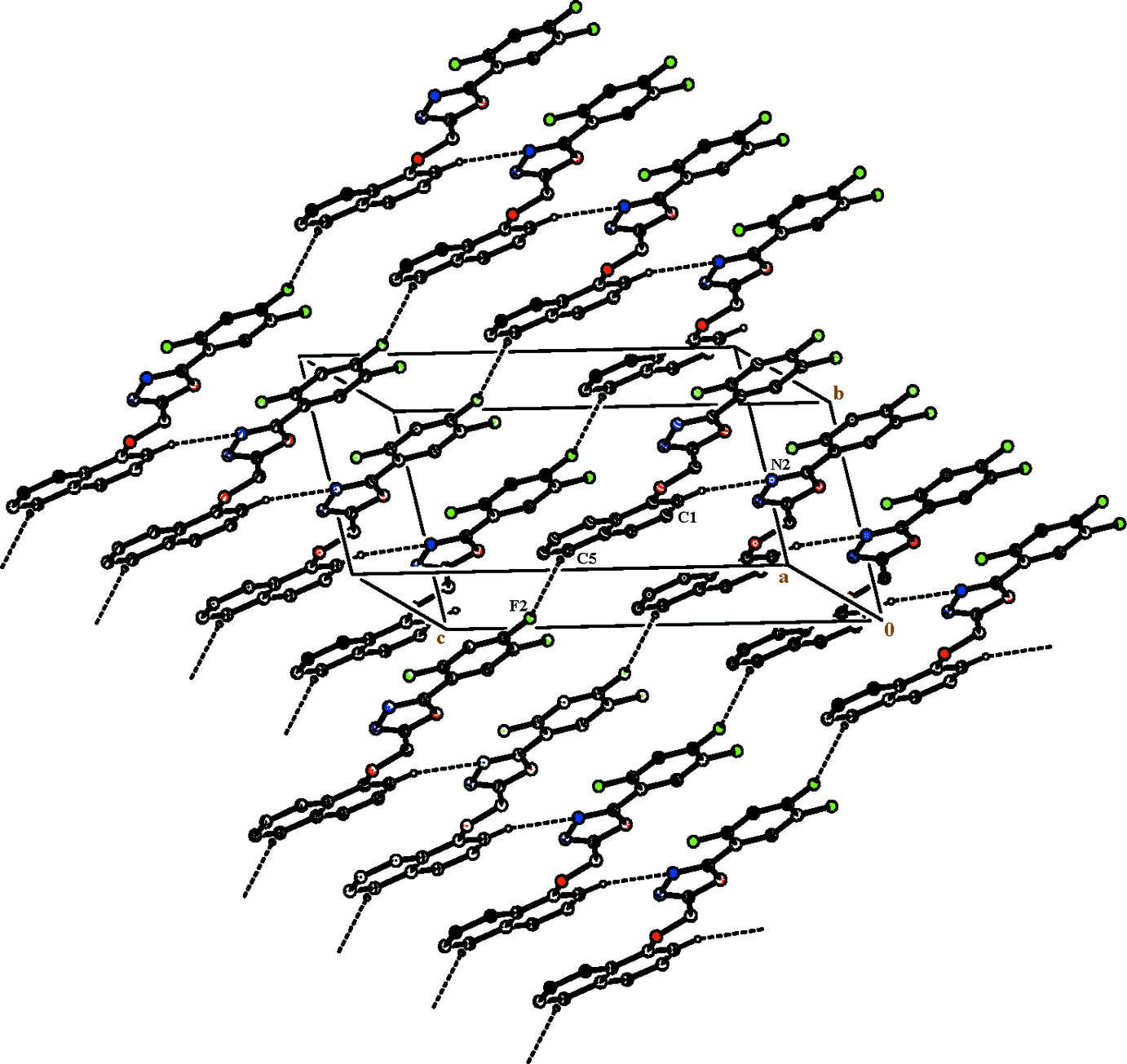

Supplement: Supplementary file 5 [file e-71-0o190-fig2.tif]
